# The non-specific lipid transfer protein N5 of *Medicago truncatula* is implicated in epidermal stages of rhizobium-host interaction

**DOI:** 10.1186/1471-2229-12-233

**Published:** 2012-12-07

**Authors:** Youry Pii, Barbara Molesini, Simona Masiero, Tiziana Pandolfini

**Affiliations:** 1 Department of Biotechnology, University of Verona, Strada le Grazie 15, Verona, 37134, Italy; 2 Department of Biology, University of Milan, Via Celoria 26, Milan, 20133, Italy

**Keywords:** *Medicago truncatula*, MtN5, Symbiosis, *Sinorhizobium meliloti*, Pre-infection stage, Root hair curling

## Abstract

**Background:**

The symbiotic interaction between leguminous plants and rhizobia involves two processes: bacterial infection, resulting in the penetration of bacteria in epidermal and cortical cells, and root nodule organogenesis. Root nodule symbiosis is activated by rhizobial signalling molecules, called Nodulation factors (NFs). NF perception induces the expression of several genes called early nodulins. The early nodulin N5 of *Medicago truncatula* is a lipid transfer protein that has been shown to positively regulate nodulation although it displays *in vitro* inhibitory activity against *Sinorhizobium meliloti*. The purpose of this work was to investigate the role of *MtN5* by studying its spatial and temporal pattern of expression during the symbiotic interaction, also in relation to known components of the symbiotic signalling pathway, and by analysing the phenotypic alterations displayed by rhizobia-inoculated *MtN5*-silenced roots.

**Results:**

We show here that *MtN5* is a NF-responsive gene expressed at a very early phase of symbiosis in epidermal cells and root hairs. *MtN5* expression is induced *in vitro* by rhizobial effector molecules and by auxin and cytokinin, phytohormones involved in nodule organogenesis. Furthermore, lipid signaling is implicated in the response of *MtN5* to rhizobia, since the activity of phospholipase D is required for *MtN5* induction in *S. meliloti*-inoculated roots. *MtN5*-silenced roots inoculated with rhizobia display an increased root hair curling and a reduced number of invaded primordia compared to that in wild type roots, but with no impairment to nodule primordia formation. This phenotype is associated with the stimulation of *ENOD11* expression, an early marker of infection, and with the down-regulation of *Flotillin 4* (*FLOT4*), a protein involved in rhizobial entry.

**Conclusions:**

These data indicate that *MtN5* acts downstream of NF perception and upstream of *FLOT4* in regulating pre-infection events. The positive effect of MtN5 on nodule primordia invasion is linked to the restriction of bacterial spread at the epidermal level. Furthermore, *MtN5* seems to be dispensable for nodule primordia formation. These findings provide new information about the complex mechanism that controls the competence of root epidermal cells for rhizobial invasion.

## Background

Plants belonging to the *Leguminosae* family have the ability to interact with rhizobia and produce a nitrogen-fixing organ, the root nodule. The symbiotic relationship starts with a molecular cross-talk between the two partners. Host-plant derived molecules are perceived by rhizobia and activate the synthesis of Nod factors (NFs), which, in turn, elicit a variety of biochemical responses in the root hair, including changes in ion fluxes, membrane depolarization, the oscillation of the cytosolic calcium and modification in the cytoskeleton [1-4] that lead to root hair deformation, to infection thread (IT) formation and eventually to the penetration of the bacteria into the epidermis [5].

NF perception relies on a pair of orthologous genes belonging to the LysM-family receptor-like kinases (LysM-RLK), NFP and LYK3 in *M. truncatula* and NFR1 and NFR5 in *L. japonicus*[6-8]. Closely related genes for NF perception were also found in other leguminous plants such as pea and soybean [9]. The perception of the NF through the LysM-RLKs activates a signalling pathway, termed the common symbiotic pathway [5], constituted in *M. truncatula* by *DMI1* (coding for an ion channel), *DMI2* (coding for a leucine-rich repeat receptor-like kinase), which are involved in generating the calcium oscillations [10-12], and *DMI3* (coding for a calcium calmodulin protein kinase), which is responsible for the decoding of calcium spiking amplitude and frequency [5]. NF-induced early infection events also involve the activity of phospholipase C and D [13,14]. NF perception and the activation of the signalling cascade take place in the root epidermis. At the same time, pericycle and cortical cells re-enter the cell cycle and form a primordium from which a nodule meristem arises [15,16]. The primordium is invaded by rhizobia harboured inside infection threads between 48 and 96 h post root hair infection.

Cell division is mainly controlled by two crucial plant phytohormones, auxin and cytokinin, which regulate the progression of cells through the cell cycle [17]. Concentrations and the auxins to cytokinins ratio both play a pivotal role in determining whether and where cells are about to enter the mitotic phase in plants [18-20]. The reduction of auxin transport in rhizobia-inoculated [18,21-23] roots changes not only the auxin fluxes but also the auxin to cytokinin ratio at the site of nodule initiation. One of the *L. japonicus* spontaneous nodulation mutants carries an alteration in the *Lotus Histidine Kinase 1* (*LHK1*) gene that acts as a cytokinins receptor [24], whilst the *LHK1* loss-of-function mutants show a marked reduction in the number of primordia and mature nodules [25]. In *M. truncatula* the RNA interference (RNAi)-mediated down-regulation of Cytokinin Response1 (*CRE1*), the *LHK1* ortholog, resulted in a marked reduction of the cortical cell division and in a block of the majority of the ITs at root hair level [26]. Thus cytokinin seems to be involved in the coordination of the epidermal and cortical pathways of nodulation most likely through the *NODULE INCEPTION* (*NIN*) gene [27,28]. Until now, *NIN* has been the supposed key gene in coordinating the NFs signalling and entry pathways [27,28].

Recent studies have suggested that membrane microdomains and associated proteins such as *Flotillin*2 (*FLOT2*), *FLOT4* and *M. truncatula SYMBIOTIC REMORIN 1* (*MtSYMREM1*), are involved in epidermal responses to rhizobia and play a role in IT formation [29,30].

The extensive analysis of the *M. truncatula* transcriptome showed that a group of small, cysteine-rich peptides are up-regulated during the establishment of symbiosis [31]. *MtN5* is a nodulin gene that was identified by means of a differential screening approach and is expressed in mature nodules [32-34]. The sequence homology suggests that MtN5 belongs to the plant non-specific Lipid Transfer Protein (nsLTP) super-family characterized by an eight cysteine motif and the phylogenetic analysis demonstrates that it has high homology with *Arabidopsis thaliana* DIR1 and groups with plant nsLTP-like protein [34,35]. Like other proteins belonging to the plant ns-LTP super-family, MtN5 is able to bind lipids *in vitro* and to inhibit the growth of pathogens and symbionts [34]. The RNAi-mediated *MtN5* suppression resulted in a marked reduction in the number of nodules developed on transgenic hairy roots suggesting that MtN5 is required for nodulation [34]. However, the stages of the nodulation pathway that *MtN5* activity might be involved in are still unknown.

The aim of this study was to gain further insight into the function of *MtN5* in the *M. truncatula*-*S. meliloti* interaction. Our data indicated that *MtN5* is a NF-responsive gene expressed at a very early phase of legume-rhizobium interaction in the root hairs and in the epidermal cells. The phenotypic analysis of *MtN5*-silenced roots showed that *MtN5* is implicated in limiting root hair curling and that this role is necessary for an efficient colonization of nodule primordia. *MtN5* response to rhizobia is dependent on phospholipase D (PLD) activity and does not seem to require *DMI1*. Furthermore, *FLOT4* induction is drastically reduced in *MtN5*-silenced roots. This study demonstrates that *MtN5* is involved in the control of rhizobial infection by acting in two apparently contrasting ways: firstly by restricting the invasion at the root epidermis and secondly by promoting the infection in the root cortex.

## Results

### Prediction of regulatory motifs in the *MtN5* promoter

Previous data demonstrated that *MtN5* is precociously induced in *S. meliloti*-inoculated roots, is expressed in the root nodules and that its function is required for the successful symbiotic interaction between *S. meliloti* and *M. truncatula*[32-35]. With the aim of gaining a deeper insight into the regulation of *MtN5* expression, the putative *MtN5* promoter was analysed by means of an *in silico* approach for the detection of conserved responsive elements.

**Figure 1:**
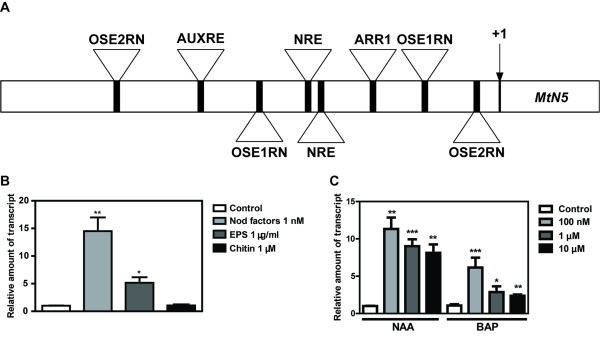
**Bioinformatic analysis of the *MtN5 *promoter and expression of *MtN5 *in *M. truncatula *roots following treatments with bacteria-derived molecules and plant hormones. A**. Schematic drawing of the putative *MtN5* promoter and *MtN5* open reading frame. The beginning of the ORF is identified by +1. The region analysed as the putative promoter encompasses 1.54 kb upstream the initiation codon. The following regulatory motifs are indicated: OSE1RN (OSE1ROOTNODULE; AAAGAT occurring in antisense orientation), OSE2RN (OSE2ROOTNODULE; AAGAG occurring in both sense and antisense orientations), NRE (AATTT), ARR1 (ARR1AT; GATT) and AUXRE (AUXREPSIAA4; GTCCCAT occurring in antisense orientation). **B**. *M. truncatula* roots were treated for 24 h with purified NFs (1 nM); for 48 h with EPS (1 μg/ml) and for 48 h with chitin (0.1 μM). **C**. *M. truncatula* roots were treated with α-naphtyl acetic acid (NAA) or with benzyl-amino-purine (BAP) at 100 nM, 1 μM and 10 μM for 48 h. The data were normalized to an internal actin control. The relative expression ratios were calculated using untreated roots as calibrator sample. The values reported are means ± SE (n=at least 3). Student’s *t* test was applied. *, P < 0.05; **, P < 0.01; ***, P < 0.001.

The sequence spanning 1.54 kb upstream of the ATG translation start codon of the *MtN5* gene (Figure 1A; Additional file 1) was analysed by using the PLACE algorithm [36,37]. Along with common elements found in promoter sequences such as TATA and CAAT boxes, the consensus sequences of the organ-specific elements (OSE) OSE1ROOTNODULE and OSE2ROOTNODULE (AAAGAT and CTCTT, respectively), which are characteristic of promoters active in infected cells of root nodules [38,39], were found in the *MtN5* promoter, although not canonically spaced (Figure 1A; Additional file 1). Moreover, the nucleotide sequence AATTT, termed Nodulation Responsive Element (NRE), was found to recur twice in the 590 bp upstream of the ATG initiation codon. This responsive element was demonstrated to be present in the promoter sequence of well characterized nodulin genes such as *MtENOD11, MtNIN* and *ERN1* and to function as a *cis*-acting element targeted by the GRAS type NSP1 transcription factor [40]. The *in silico* analysis of the *MtN5* promoter also highlighted the presence of motifs involved in the hormonal control of gene expression such as ARR1AT and AUXREPSIAA4, responsive to cytokinin and auxin respectively [41,42]. Interestingly the auxin response element AUXREPSIAA4 had been previously shown to be tissue specific and characteristic of those genes that are expressed in the root apical meristem of pea plants [42].

### *MtN5* expression is induced by both rhizobia-derived molecules and plant hormones

On the basis of the motifs predicted by the PLACE algorithm [36], *MtN5* expression was analyzed after treating the roots with both microorganism elicitor molecules and phytohormones. *MtN5* expression is up-regulated after treatment with *S. meliloti* NFs [32 and this work], exhibiting an approximately 15-fold increase in NF treated roots compared with untreated roots (Figure 1B). However, a transient induction of *MtN5* transcript level was also observed after inoculation with a *S. meliloti* strain defective in NF production [32]. To test whether other rhizobia-derived signals potentially contribute to *MtN5* induction, we tested *MtN5* expression in roots treated with exopolysaccharides (EPS) extracted from *S. meliloti*[43]. The roots were also treated with molecules like chitin oligomers (N,N’,N”,N”’-tetraacetylchitotetraose) that can be originated from the degradation of fungal cell wall [44]. In roots treated with 1 μg/ml EPS, the *MtN5* transcript level increased about 5-fold as compared to the level in untreated roots, whilst *MtN5* expression was not affected by chitin treatment (0.1 μM) (Figure 1B).

Considering that bioinformatic analysis of the *MtN5* promoter predicts the presence of elements responsive to auxins and cytokinins, the phytohormones involved in the regulation of root nodules initiation and growth, the effects of auxin and cytokinin treatments on root *MtN5* expression were evaluated. As reported in Figure 1C, both α-naphthyl acetic acid (NAA) and benzyl-amino-purine (BAP) supplied to roots can induce *MtN5* expression in a wide range of concentrations (*i.e.* from 100 nM to 10 μM). Auxin displayed a similar ability to stimulate *MtN5* expression at each of the concentrations tested whilst the effect of cytokinin was more pronounced at the lowest concentration used (100 nM) (Figure 1C). Interestingly, a stimulatory effect of auxin and cytokinin on *MtN5* expression was detected in *M. truncatula* Jemalong *in vitro*-cultured leaf explants after a combined treatment with NAA and BAP [45,46].

The *in silico* analysis of the *MtN5* promoter sequence and the experimental evidence suggest that *MtN5* primarily responds to rhizobia-derived signals and can be regulated by hormones involved in the coordination of epidermal and cortical responses and in nodule formation.

### *MtN5* expression pattern during rhizobial infection and nodule development

To investigate the spatial and temporal pattern of *MtN5* expression at the tissue and cell levels, *MtN5* promoter activity was monitored in *MtN5p::GUS* transgenic roots with and without rhizobial inoculation. In non-inoculated roots, *MtN5p::GUS* is expressed at the root tip (Additional file 2). The β-glucuronidase (GUS) activity was also detected at the site of lateral root emergence and along the whole length of young lateral roots; as lateral roots get older, GUS activity was confined to the root tip (Additional file 2).

**Figure 2:**
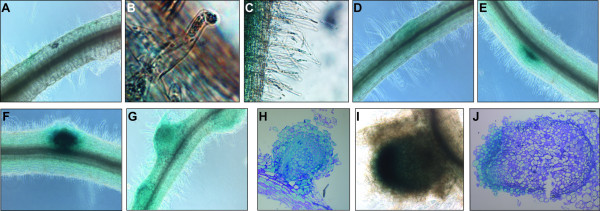
***MtN5 *promoter activity during rhizobial infection and nodule development.** Representative expression patterns are shown*.* Localization of *MtN5* expression in the root epidermis (**A**) and in the root hairs (**B**, **C**) 3 hours post-inoculation (hpi). (**D**) GUS staining detected in the root cortex at 24 hpi. *MtN5* promoter activity in nodule primordia (**E**, **F**) and in young nodules (**G**, **H**). *MtN5* promoter activity in fully developed root nodules (**I**, **J**).

*MtN5p::GUS* transgenic roots showed localized induction of GUS activity as a consequence of *S. meliloti* inoculation. 3 hours post-inoculation (hpi), GUS activity was visible in the epidermis as localized spots and in the root hairs (Figure 2A-2C). At more advanced stages of infection (*i.e.* 24 hpi), the *MtN5* promoter activity was detected in the root cortex, in close proximity to the central stele (2D).

During the early stages of nodule development, considerable activity of the *MtN5* promoter could be observed at the boundary between the root cortex and the central stele (nodule primordia) (Figure 2E). As primordia grow and emerge from the root, the expression of the GUS reporter is detectable in the whole nodule (Figure 2F-2H). In fully developed root nodules, the promoter activity was predominantly localized in the distal zone (Figure 2I and 2J). The analysis of *MtN5* promoter activity highlighted the presence of *MtN5* expression during rhizobial infection in those cells that underwent structural changes and membrane rearrangements (*i.e.* root hairs) and in tissues showing a high cell division activity such as root meristems and nodule primordia.

The temporal and spatial pattern of *MtN5* promoter activity indicates that *MtN5* can be considered as an early marker of *M. truncatula* and *S. meliloti* interaction together with other epidermal nodulins such as *RIP1* and *ENOD11,* which are putatively involved in cell wall modification before the formation of infection threads [47,48].

### *MtN5* influences *ENOD11* response to rhizobia and is not required for *NIN* induction

**Figure 3:**
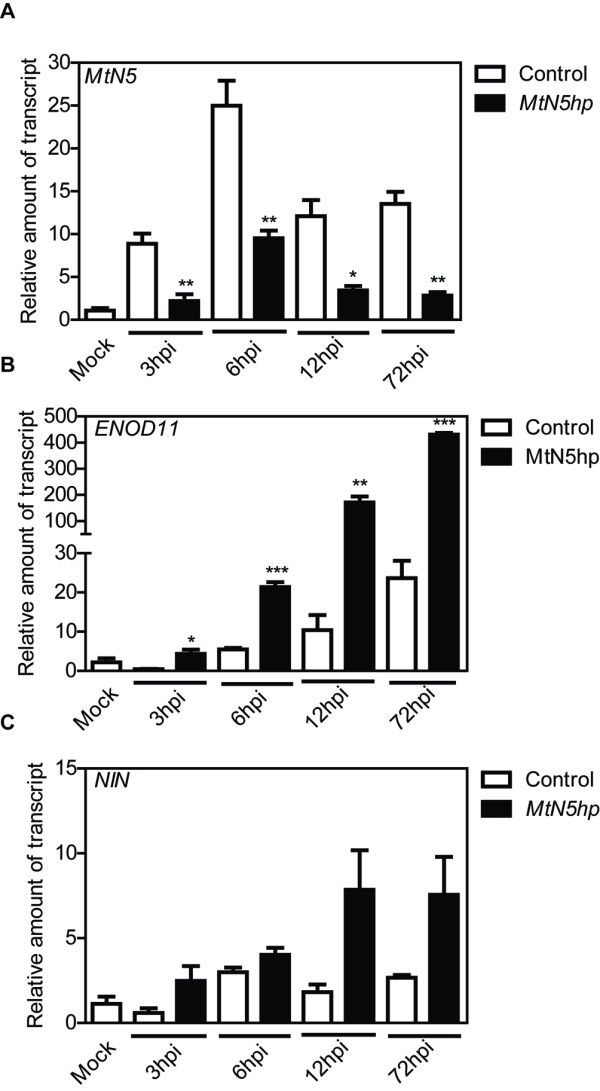
**Time course expression analysis of *MtN5, ENOD11 *and *NIN *genes after rhizobial inoculation.** The expression level of *MtN5* (**A**), *ENOD11* (**B**) and *NIN* (**C**) was assessed by qRT-PCR in adventitious hairy roots obtained by genetic transformation with *A. rhizogenes* harbouring a pRedRoot binary vector carrying either the *MtN5hp* construct or an empty T-DNA. The data were normalized to an internal actin control. The relative expression ratios were calculated using control mock-inoculated roots as calibrator sample. The values reported are means ± SE (n=at least 3). Student’s *t* test was applied. *, P < 0.05; **, P < 0.01; ***, P < 0.001.

In order to understand the relationships between *MtN5* and other components of the early NFs signalling pathway, the expression of two early nodulins, *ENOD11* and *NIN* was analysed in transgenic adventitious hairy roots carrying a hairpin construct (*MtN5hp*) for *MtN5* silencing [34]. Composite plants were micro-flood inoculated with *S. meliloti* and roots were collected at different times after infection (3, 6, 12, and 72 hpi). The roots considered in this experiment were preselected for their transgenic status on the basis of the fluorescent signal deriving from the DsRED marker gene present in the T-DNA. *MtN5* expression was induced very rapidly after *S. meliloti* inoculation (Figure 3A) in adventitious control roots, which were generated from the infection with *Agrobacterium rhizogenes* carrying an empty pRedRoot vector. In inoculated *MtN5hp* transgenic roots, *MtN5* expression was significantly reduced at each time point considered compared to that in control roots (Figure 3A). The steady state level of the *MtN5* transcript decreased on average by 70%, ranging from approximately 62% (6 hpi) to 80% (72 hpi).

The expression of *ENOD11,* an early marker of pre-infection and infection phases of rhizobial symbiosis, showed a progressive increase starting from 6 hpi in the control adventitious roots. In *MtN5*-silenced root tissues, *ENOD11* was already strongly up-regulated at 3 hpi and its expression was significantly enhanced as compared to that in control roots in the following stages of infection (Figure 3B).

*MtNIN* is required for nodule primordia initiation having a role in the coordination of epidermal and cortical responses [28]. In control roots the expression of *MtNIN* was up-regulated starting from 6 hpi. *MtN5* silencing did not significantly alter *NIN* mRNA steady state levels at each time point examined in the analysis (Figure 3C).

**Figure 4:**
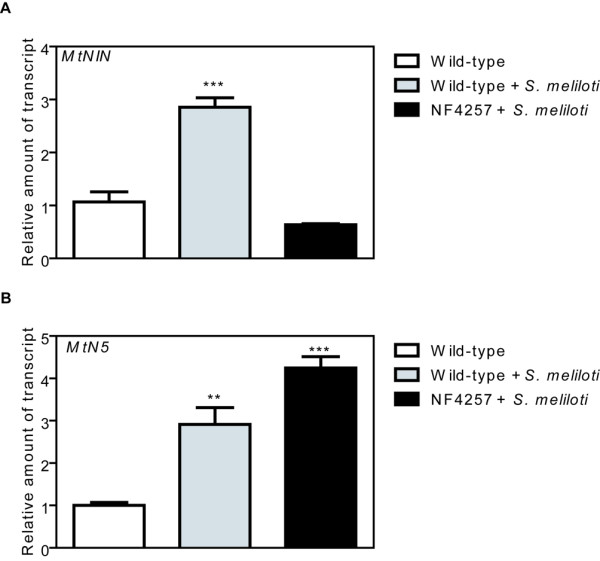
**Expression level of *NIN *and *MtN5 *genes in *M. truncatula DMI1 *insertional mutant line.** The expression level of *NIN* (**A**) and *MtN5* (**B**) was assessed in *M. truncatula* R108 wild-type and *DMI1* insertional mutant roots, both mock-inoculated and inoculated with *S. meliloti*. The data were normalized to an internal actin control. The relative expression ratios were calculated using mock-inoculated roots as calibrator sample. The values reported are means ± SE (n=3). Student’s *t* test was applied. **, P < 0.01; ***, P < 0.001.

To date, the data available regarding the involvement of nodulin genes in the NFs signalling pathway place both *ENOD11* and *MtNIN* downstream of the calcium spiking [13,28]. To analyze the relationship between *MtN5* expression and the calcium oscillation we used the recently established *Tnt1* transposon mutant collection of *M. truncatula* R108 [49] and searched for an insertion line in *DMI1* gene, which is required for the generation of calcium spiking [10,50]. We identified the line NF4257, which carries a transposon insertion 155 nucleotides downstream of the translation initiation site of *DMI1* gene. Plants were propagated and were subsequently screened for homozygous offsprings by polymerase chain reaction (PCR) (Additional file 3). In the NF4257 homozygous line, *DMI1* expression was almost completely abolished (*DMI1* steady state level was 20% as compared with wild type plants) (Additional file 3). In the *S. meliloti*-inoculated NF4257 mutant, the *MtNIN* induction was eliminated (Figure 4A and [28]), whilst *MtN5* showed a wild-type behaviour (Figure 4B). This finding suggests that *MtN5* activation might occur upstream of calcium spiking, although we cannot exclude a priori that other *Tnt1* insertions, potentially present in the *DMI1* homozygous mutant seedlings, could affect *MtN5* expression in *trans*.

### PLD activity is required for the response of *MtN5* to *S. meliloti*

Several pieces of research have demonstrated that the signalling cascade activated by NFs implicates, at an early stage, the intermediation of heterotrimeric G-proteins and small GTPases [51-54], which, in turn, stimulate the functionality of phospholipase C (PLC) and D (PLD) [51,53,55,56]. According to the current model, the products of hydrolysis generated by PLC (inositol trisphosphate and diacyl glycerol) stimulate the activity of ligand-gated calcium pumps causing the increase in the cytosolic Ca^2+^ concentration, whereas the PLD product (phosphatidic acid, PA) seems to be required for the onset of a kinase/phosphatase signalling cascade, that eventually leads to the activation of nodulin genes and to the reorganization of the cytoskeleton in preparation for the root hair inward growth [57].

**Figure 5:**
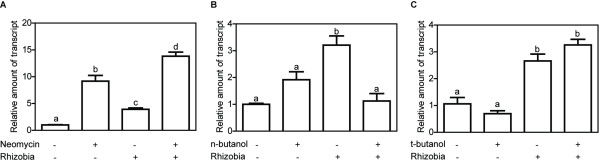
**Effect of specific inhibitors of PLC and PLD (neomycin and n-butanol, respectively) on *MtN5 *expression. A**. The relative mRNA level of *MtN5* was assessed by qRT-PCR analysis in wild-type *M. truncatula* roots either untreated or treated with PLC agonist neomycin and inoculated with *S. meliloti*. **B**. qRT-PCR performed on wild-type *M. truncatula* roots either untreated or treated with PLD agonist n-butanol and inoculated with *S. meliloti*. **C**. qRT-PCR carried out on wild-type *M. truncatula* roots either untreated or treated with n-butanol isomer, t-butanol, and inoculated with *S. meliloti*. The expression data were normalized to an internal actin control. The relative expression ratios were calculated using untreated, mock-inoculated roots as calibrator sample. The data reported are means ± SE (n = at least 3). Common letters indicate no significant difference according to one-way ANOVA with the Bonferroni post-test.

With the aim of investigating the dependence of *MtN5* induction on the two parallel pathways of the lipid signalling, a pharmacological approach was adopted, using specific inhibitors of PLC and PLD (neomycin and n-butanol, respectively). As already demonstrated, the application of both 100 μM neomycin and 68 mM n-butanol did not cause a significant loss of viability in root hair cells [13]. *M. truncatula* seedlings, pretreated with neomycin and subsequently inoculated with *S. meliloti*, showed a 13-fold up-regulation of *MtN5* (Figure 5A) compared to untreated, control roots, whereas in the absence of the pharmacological treatment, inoculated roots displayed a 4-fold *MtN5* induction indicating that neomycin did not repress *MtN5* induction. Similarly, the treatment with the PLC inhibitor alone resulted in an approximately 9-fold increase of *MtN5* expression. Analogous stimulatory effects were reported for other nodulin genes after treatment with PLC inhibitors and interpreted as the result of membrane trafficking alteration or microtubule reorganization [14,58]. These data suggest that the *MtN5* induction due to rhizobial inoculation and to neomycin treatment are most probably independent effects (Figure 5A). The n-butanol competes for the phosphatidyl group acting as antagonist of PA production by PLD enzymatic activity [59]. Interestingly, *S. meliloti*-inoculated roots treated with the PLD inhibitor did not show a significant variation in *MtN5* expression when compared to control roots indicating that the n-butanol treatment prevents *MtN5* induction (Figure 5B). The treatment with tert-butanol, a butanol isomer, which does not act as a phosphatidyl group acceptor, did not hamper *MtN5* induction (Figure 5C). On the whole, these results suggest that *MtN5* response is dependent on PA production.

### *MtN5* influences root hair curling and primordia invasion

**Figure 6:**
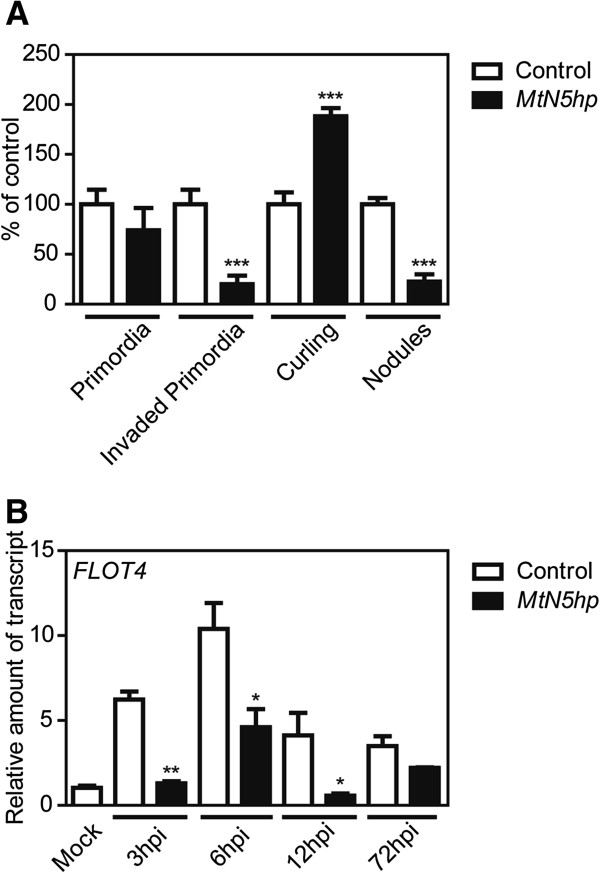
**Phenotypical analysis of inoculated *MtN5hp *roots and *FLOT4 *expression. A**. Number of root hair curling events, total and invaded primordia and mature nodules in *MtN5hp* roots inoculated with *S. meliloti*. The data reported are means ± SE (n=40) calculated as percentage relatively to control inoculated roots. Student’s *t* test was applied. ***, P < 0.001. **B**. The expression level of *FLOT4* was assessed by qRT-PCR in adventitious hairy roots obtained by genetic transformation with *A. rhizogenes* harbouring a pRedRoot binary vector carrying either the *MtN5hp* construct or an empty T-DNA (control). The expression data were normalized to an internal actin control. The relative expression ratios were calculated using control mock-inoculated roots as calibrator sample. The values reported are means ± SE (n=3). Student’s *t* test was applied. *, P < 0.05; **, P < 0.01.

The microscopic observations of *MtN5hp* hairy roots inoculated with a rhizobium strain harbouring the *hemA::LacZ* reporter gene revealed a significantly higher number - about twice that of control roots - of root hair curling events (Figure 6A). However, the 5-bromo-4-chloro-3-indolyl-beta-D-galactopyranoside (X-Gal) staining of rhizobia highlighted that the number of bacteria-colonized primordia was significantly reduced (by about 80%) in *MtN5hp* roots as compared to that in the control samples (Figure 6A) and, as already reported, the composite plants bearing *MtN5hp* silenced roots also showed a reduction in the number of fully developed nodules (by about 80%). Nonetheless, the total number of nodule primordia did not vary significantly between *MtN5*-silenced and control roots (Figure 6A) nor were any phenotypic alterations observed in curled root hairs such as excessive curling or root hair deformation (Additional file 4). It was also apparent that nodule primordia and mature nodules of *MtN5hp* plants were not dissimilar in morphology to those produced in control roots (Additional file 4). On the basis of these data, the transcript levels of *M. truncatula Flotillin 4* (*FLOT4*), a gene involved in the bacterial entry pathway, were compared in *MtN5-*silenced roots and control roots. *FLOT4* showed itself to be readily induced in control roots after rhizobial inoculation (3 hpi), reached a maximum of expression at 6 hpi and decreased afterwards. In *MtN5hp* roots infected with the symbiont, a drastic decrease of *FLOT4* expression (between 70% and 90%) was detected between 3 and 12 hpi (Figure 6B).

## Discussion

The early nodulin *MtN5* was identified by means of a subtractive hybridization approach [32] and, according to the sequence homology, was annotated as a putative Lipid Transfer Protein [32,33]. The *in vitro* characterization of MtN5 recombinant protein highlighted that it has the ability to bind lipid and counteract the growth of microorganisms [34] suggesting a possible involvement in the limitation of the rhizobial invasion. However, the functional study based on both silencing and over-expression approaches showed that *MtN5* function is required for a successful establishment of the symbiosis [34].

To shed light on the molecular role of *MtN5* in *M. truncatula-S. meliloti* symbiosis, we initially focused on the regulation of *MtN5* expression. The data presented in this work confirm that the induction of *MtN5* is a NF-responsive event, but also show that *MtN5* expression is probably controlled by several additional signals, either rhizobia-derived effectors or phytohormones, auxin and cytokinins. These observations are consistent with the finding that *MtN5* is induced at different stages of symbiotic interaction from pre-infection to mature nodules and also with the constitutive expression in root tips of principal and lateral roots. The rhizobial infection does not modify *MtN5* expression at the root tip level, but it triggers very early the induction of *MtN5* in the epidermal tissues (3 hpi) and in the root cortex (24 hpi). Thus we propose that *MtN5* can be regarded as an early molecular marker of *M. truncatula* and *S. meliloti* interaction.

The detection of *MtN5* promoter activity in root hairs and in localized spots of the epidermis supports the idea that *MtN5* might be involved in pre-infection or/and infection events. Pre-infection responses to rhizobia are accompanied by the induction of the marker gene *ENOD11* in the root epidermis, whereas in later phases its expression is confined to the invaded zones of the roots [48]. The transcription factor *MtNIN* seems to regulate *ENOD11* activity contributing to the restriction of its expression to the *S. meliloti*-responsive zone of the root [28]. In inoculated *MtN5*-silenced roots, the *ENOD11* induction is not prevented. Indeed, *ENOD11* expression is strongly stimulated in *MtN5*-silenced roots compared with that in control roots, indicating that *MtN5* function is required for controlling *ENOD11* expression. In addition, *MtN5* down-regulation does not inhibit *NIN* transcription suggesting that the mis-regulation of *ENOD11* in *MtN5*-silenced roots is independent of the *NIN* effect on *ENOD11* transcription. The stimulation of *ENOD11* expression in *MtN5*-silenced roots could also be interpreted as an indirect consequence of the impaired rhizobial infection.

The phenotypic analysis of *MtN5*-silenced roots confirms a role of this nodulin in pre-infection stages of the symbiosis; in fact, although abnormalities in root hair curling were not observed, the number of curled root hairs was significantly greater in comparison to that in control roots. An enhanced responsiveness of root hairs to rhizobia was observed in several infection-defective leguminous plants [28,60-62], which could suggest the presence of a mechanism that represses root cell competence in wild type plants [61]. From our data we can infer that *MtN5* might be implicated in the mechanism that limits root hair curling.

The host plant controls the rhizobial invasion with different strategies [63,64]. For instance, the induction of pathogen defence genes is one of the early events detected during symbiosis and it has been indicated as one of the mechanisms involved in IT abortion [33,65,66]. At later stages, the expression of these defence genes usually declines probably as a consequence of bacteria recognition by the host plant mediated also by molecular signals, e.g. exopolisaccharides, produced by rhizobia [33,65,67]. Furthermore, host plants limit the number of nodules through a mechanism termed autoregulation of nodulation that implicates the intervention of shoot receptor kinases [63,64]. MtN5 belongs to the non specific LTP family that includes apoplastic proteins with inhibitory activity against pathogens and itself possesses the capacity to limit *S. meliloti* growth *in vitro*[34]. It has also been suggested that MtN5 could belong to a group of LTPs having a role in plant-microbe interaction signalling [35]. MtN5 might act by limiting the stable attachment and/or proliferation of rhizobia at the surface of epidermal cells/root hairs, by modulating, for instance, either the perception or the activity of rhizobia-derived signal molecules. Besides the increased curling, *MtN5*-silenced roots showed a reduction in the number of invaded root nodules, indicating that *MtN5* could also participate, either directly or indirectly, in the regulation of bacterial invasion. If this is so, the limitation of curling events might favour the penetration of bacteria at a restricted number of infected spots.

Several experimental observations are consistent with the existence of a parallel NF-mediated signalling pathway that operates in root hair curling and bacterial entry acting in concert with the common symbiotic pathway [25,68]. Furthermore, pharmacological studies have demonstrated that lipid signalling is implicated in both root hair deformation and IT initiation [14]. The response of *MtN5* to rhizobium is dependent on PLD activity and we obtained a first indication that it might be independent of *DMI1*, a component of the common symbiotic pathway. Further investigations are needed to prove the independence of *MtN5* induction from Ca^2+^ spiking and to ascertain whether *MtN5* is a component of the parallel NF-mediated pathway that controls root hair curling and/or rhizobial entry. It was recently discovered that a member of the flotillin family, *FLOT4,* which is up-regulated during early symbiotic events and localized in membrane microdomains in infected root tips, plays a role in IT initiation and elongation [29]. Our observations indicate that *MtN5* acts upstream with respect to *FLOT4*, supporting the idea that *MtN5* participates in the events preceding IT development.

Bacterial infection occurring at the epidermis level is coordinated with the cortical process that promotes nodule formation and development [19]. The formation of nodule primordia requires the perception of Ca^2+^ oscillations by calcium-activated kinase CCaMK (DMI3) and the activation of cytokinin signalling. In addition, the phytohormone auxin participates both locally and systemically in the regulation of nodule organogenesis [22,69]. Both auxin and cytokinins induce *MtN5* expression *in vitro*. Furthermore, in *S. meliloti* inoculated roots the *MtN5* transcript localizes in the inner cortex at a site where cellular divisions take place to form the nodule primordium. These findings would suggest the possibility that *MtN5* participates in nodule organogenesis. However, we observed that the total number of nodule primordia in *MtN5*-silenced roots did not differ from that measured in wild type roots. Therefore, *MtN5* does not seem to be required for nodule primordia initiation. This is consistent with the finding that *MtN5* functions downstream or more likely independently of *MtNIN* (Figure 3C).

Even though nodule initiation appears not to be impaired, the number of invaded primordia was markedly reduced in *MtN5*-silenced roots. Thus the decreased number of mature nodules in *MtN5*-silenced roots could be the consequence of the curtailed capacity of the rhizobia to invade the primordia. It has in fact been reported that bacterial invasion can be crucial for the maintenance of nodule developmental program [70]. On the other hand, we cannot exclude that *MtN5* might have a direct role in promoting the maintenance of nodule primordia. Further investigations are necessary to clarify this point.

## Conclusions

The data presented here demonstrate that *MtN5* participates in the molecular events occurring at the epidermis after NF perception and PLD activation and before root hair invasion and that its function is dispensable for nodule initiation but required for nodule invasion. Plants lipid transfer proteins are usually small, secreted, basic proteins, characterized by the presence of a hydrophobic cavity that enables the interaction with lipid molecules. Members of the plant LTP superfamily display a wide range of biological activities such as a defensive role against pathogens, deposition of cuticular wax, modification of cell walls and pollen tube guidance [71,72]. Based on sequence similarity, MtN5 and *A. thaliana* DIR1, an nsLTP implicated in pathogen defence systemic signalling [73] have been assigned to a sub-group of ns-LTP putatively involved in lipid-mediated signalling [35]. The lily LTP SCA and the *A. thaliana* SCA, which are implicated in pollen tube growth, have also been put forward as signal transducers [72,74].

We propose that MtN5 plays a role in the process that regulates the competence of epidermal cells for rhizobial infection. Thus MtN5 would be part of the machinery that gives the host control over the mutualistic partner in order to preserve plant fitness. To explore the hypothesis that MtN5 functions as a signal transducer in these processes, the identification of MtN5 interacting proteins or ligands would be necessary.

## Methods

### Bacterial strains

*Sinorhizobium meliloti* 1021 [75] was grown at 28°C in LBMC medium (10 g/l tryptone, 5 g/l yeast extract, 10 g/l NaCl, 2.6 mM MgSO_4_, 2.6 mM CaCl_2_) supplemented with streptomycin 200 μg/ml. *Agrobacterium rhizogenes* ARqua1 [76] was grown at 28°C in TY medium (5 g/l tryptone, 3 g/l yeast extract, 6 mM CaCl_2_, pH 7.2) supplemented with streptomycin 100 μg/ml.

### Plant growth and rhizobial inoculation

*Medicago truncatula* cv. *Jemalong* seeds were sterilized and germinated as already described [77]. For time course assay and for GUS detection experiments, *M. truncatula* seedlings were placed in square Petri plates, containing slanted BMN agar medium [78] supplemented with 0.1 μM L-α-2-Aminoethoxyvinyl glycine (AVG). The plates were kept vertically in a growth chamber at 25°C and 10-h light/14-h dark regimen. After seven days of nitrogen starvation, the seedlings were micro-flood inoculated as previously described [34]. Briefly, bacteria were grown overnight and suspended in 10 mM MgSO_4_. Microflood inoculation was performed by placing five drops (0.5 μl) of bacterial suspension on the surface of the root. For qRT-PCR experiments, control samples (mock-inoculated) were treated with the same volume of 10 mM MgSO_4_.

### GUS constructs and histochemical staining

The reporter construct was prepared by fusing together the *MtN5* promoter and the intronless coding region of *E. coli* β-glucuronidase (*uidA*), as already described [79]. Briefly, 1.5 kb-long sequence upstream of the transcription initiation site was amplified from wild-type plants by means of PCR with the following primers: 5^′^-GAATTCCACAATCTCTTTCTTTCTCG-3^′^ and 5^′^-GGATCCCTGGTTCTAGTTTACTATAT-3^′^. The PCR fragments were sub-cloned and checked by sequencing. The *MtN5* promoter was cloned upstream the 1.812 kb sequence of GUS coding region into a pBIN19 derivative vector, harbouring the *nptII* gene, coding for the kanamycin resistance, under the transcriptional control of *nos* promoter [80]. The two transcriptional cassettes (*MtN5promoter::GUS* and kanamycin resistance) were placed convergently. The resulting chimeric gene was mobilized into *A. rhizogenes* ARquaI, which was used to obtain plants bearing genetically modified silenced roots. The histochemical GUS staining was performed as previously described [81]. Images were taken with a Leica DM2500 microscope equipped with a DFC420C digital camera (Leica Microsystems, Wetzlar, Germany). The GUS stained nodules were embedded, after fixation and dehydratation, in Technovit 7100 (HeraeusKulzer, Wehrheim, Germany) according to the manufacturer’s instructions. Sections (6 μm thick) were prepared and stained with 0.05% toluidine blue. The slides were observed with a Zeiss Axiophot D1 microscope (http://www.zeiss.com/) and images were recorded with an Axiocam MRc5 camera (Zeiss) using the Axiovision program (version 4.1).

Plants inoculated with *S. meliloti* bearing the pXLGD4 plasmid, containing the constitutive *hemA::LacZ* gene, were stained as previously described [82]. Whole root samples were mounted on glass slides with coverslips and observed with a Leica DM2500 microscope equipped with a DFC420C digital camera (Leica Microsystems, Wetzlar, Germany).

### Plant transformation

Root transformation with *A. rhizogenes* ARqua1 was performed as previously described [83]. Plants infected with ARqua1 were kept in square Petri dishes containing Fåhraeus Modified Medium (FMM) for about three weeks. When the binary vector employed in the transformation was pBIN19 (*MtN5p::GUS* construct), the FMM was supplemented with kanamycin 50 μg/ml for transformants selection. For *MtN5* silencing, the *MtN5hp* construct was cloned into the pRedRoot binary vector, as previously described [34]. Transformed roots were checked using a Leica MZ16F fluorescence microscope with the following filter setting for DsRED1 detection: 541-551 nm bandpass excitation filter and 590 nm long-pass emission filter. Composite plants were micro-flood inoculated in Petri dishes and kept vertically in the growth chamber at 25°C and 10-h light/14-h dark regimen, as previously described [34].

### Exopolysaccharide extraction

The extraction of exopolysaccharide (EPS) from *S. meliloti* 1021 was performed as previously described [43]. The polysaccharides were dissolved in 10% NaCl and the concentration of the preparation was checked using the phenol-H_2_SO_4_ method [84].

### Plant treatments

To test the effects of plant hormones on *MtN5* gene expression, *M. truncatula* seeds were scarified and sterilized as described above and then germinated on solid FMM medium. 7-day-old seedlings were moved to square Petri dishes containing slanted FMM agar medium supplemented with either α-naphthyl acetic acid (NAA) or benzyl-amino-purine (BAP) at different concentrations. Plants were kept vertically in the growth chamber at 25°C with a regimen of 10 h of light and 14 h of darkness for 48 h. Root apparatuses were collected, frozen in liquid nitrogen and stored at -80°C until RNA extraction.

For the treatments with Nod Factors (NFs), chitin tetramers (N,N’,N”,N”’-tetraacetylchitotreaose) (Carbosynth, UK) and exopolysaccharides (EPS), 7-day-old seedlings were transferred to 50 ml test tubes containing liquid FMM supplemented with the chosen concentration of the effector and kept in the growth chamber at 25°C with a regimen of 10 h of light and 14 h of darkness. The treatment with NFs was carried out for 24 h and the treatments with EPS and chitin tetramers were performed for 48 h.

For the pharmacological treatments seven-day-old *M. truncatula* seedlings were placed on Petri dishes containing BMN medium supplemented with both 0.1 μM AVG and the pharmacological effectors, and treated for 24 h. Neomycin (Sigma) was prepared as 10 mM aqueous solution and n-butanol and tert-butanol were diluted in sterile water just before use [13]. The plants were then transferred onto fresh BMN medium supplemented only with AVG and micro-flood inoculated with *S. meliloti*, as previously described. The root tissues were collected 4 hpi and *MtN5* expression was checked by quantitative RT-PCR (qRT-PCR) analysis.

### Quantitative RT-PCR

The qRT-PCR analyses were carried out as already described [85]. The nucleotide sequences of the primers used for the qRT-PCR are reported in Additional file 5. The pairs of primers used to analyse the expression of *MtN5* in hairy roots were specifically chosen at the 3^′^ end of the transcript to avoid the amplification of sequences derived from the *hp* construct itself.

### Genomic DNA extraction and insertional mutant characterization

The *M. truncatula* genomic DNA was prepared from 100 mg of leaves as previously described (http://medicago.org/documents/Protocols/dna.html).

The genetic characterization of the *M. truncatula* R108 NF4257 mutant line which, besides other insertions, harbours a transposon insertion in *DMI1* gene, was carried out by means of a PCR-based approach. The genomic DNA was used as template in a PCR reaction containing two primers annealing on the *DMI1* coding sequence (DMI1_for: 5^′^-ATCCTTGGCTGGAGTGACAAATTG-3^′^; DMI1_rev: 5^′^-CTGATCTGCATTTTCGTCCGCAGC-3^′^) and a third primer complementary to the *Tnt1* sequence (Tntail1: 5^′^-TATGCAAAGAACTTGTCGGCATGC-3^′^) [49] (Additional file 3). The discrimination between heterozygous and homozygous plants for the insertion in the *DMI1* gene was carried out on the basis of the number and the size of the amplicons obtained following PCR reaction.

### Statistical analysis

The mean values ± SE are reported in the figures. Statistical analyses were conducted using a Student’s *t* test or a one–way ANOVA with Bonferroni post-test, as appropriate.

## Competing interests

The authors declare that they have no competing interests.

## Authors’ contributions

YP carried out the nodulation experiments and wrote the manuscript; YP, BM performed the molecular analyses; SM performed the microscopic analysis of the root nodules; TP coordinated the study and wrote the manuscript. All authors read and approved the final manuscript.

## Supplementary Material

Additional file 1 **Nucleotide sequence of the putative *MtN5 *promoter and *MtN5 *open reading frame.** The beginning of the ORF is identified by +1. The region analysed as the putative promoter encompasses 1.54 kb upstream the initiation codon. TATA box and CAAT box are double underlined and underlined with dashed line, respectively. The other motifs highlighted are listed as follow: OSE1ROOTNODULE (AAAGAT occurring in antisense orientation), OSE2ROOTNODULE (AAGAG occurring in both sense and antisense orientations), NRE (AATTT) , ARR1AT (AGATT) and AUXREPSIAA4 (GTCCCAT occurring in antisense orientation). Click here for file

Additional file 2 ***MtN5 *promoter activity in non-inoculated *M. truncatula *root tissue.** A. Representative GUS staining pattern in *M. truncatula* transgenic adventitious roots harbouring the *MtN5::GUS* construct. Insets show the representative GUS staining pattern at the lateral root apex (B) and at the lateral root emergence (C), respectively. Click here for file

Additional file 3 **Description of NF4257 insertional mutant and determination of *DMI1 *expression in the mutated background.** A. Schematic drawing of the *Tnt1* insertion and representation of the oligonucleotides used for the genetic characterization of the insertional line. B. qRT-PCR analysis of *DMI1* expression in *M. truncatula* R108 wild-type and NF4257 roots inoculated with *S. meliloti*. The expression data were normalized to an internal actin control. The relative expression ratio was calculated using inoculated wild type roots as calibrator sample. The values reported are means ± SE (n=3). Student’s t test was applied. **, P < 0.01. Click here for file

Additional file 4 **Root hair deformations and nodule primordia in *MtN5-*silenced and control roots.** A-B. Representative micrographs of root hair curling in *M. truncatula* roots inoculated with *S. meliloti.* A. Transgenic adventitious root bearing the *MtN5hp* gene construct. B. Transgenic adventitious root bearing an empty T-DNA (Control sample). C-D. Representative micrographs of root nodule primordia in *M. truncatula* roots inoculated with *S. meliloti* carrying the pXLGD4 plasmid*.* The presence of rhizobia within the primordia was highlighted through the staining for the β-galactosidase activity. C. Nodule primordium generated on transgenic adventitious root bearing the *MtN5hp* gene construct. D. Nodule primordium generated on transgenic adventitious root bearing an empty T-DNA (Control sample). Click here for file

Additional file 5 **Primers used for RT-PCR.** List of the oligonucleotides used as primers in the qRT-PCR experiments. Click here for file

## References

[B1] Timmers ACJ Auriac MC Truchet G Refined analysis of early symbiotic steps of the Rhizobium-Medicago interaction in relationship with microtubular cytoskeleton rearrangements Development 1999 126 3617 3628 10409507 10.1242/dev.126.16.3617

[B2] Sieberer BJ Timmers AC Emons AM Nod factors alter the microtubule cytoskeleton in Medicago truncatula root hairs to allow root hair reorientation Mol Plant Microbe Interact 2005 18 1195 1204 10.1094/MPMI-18-1195 16353554

[B3] Cárdenas L Thomas-Oates JE Nava N López-Lara IM Hepler PK Quinto C The role of nod factor substituents in actin cytoskeleton rearrangements in Phaseolus vulgaris Mol Plant Microbe Interact 2003 16 326 334 10.1094/MPMI.2003.16.4.326 12744461

[B4] de Ruijter NCA Bisseling T Emons AMC Rhizobium Nod factors induce an increase in subapical fine bundles of actin filaments in Vicia sativa root hairs within minutes Mol Plant Microbe Interact 1999 12 829 832 10.1094/MPMI.1999.12.9.829

[B5] Oldroyd GE Downie AJ Coordinating nodule morphogenesis with rhizobial infection in legumes Annu Rev Plant Bio 2008 59 519 546 10.1146/annurev.arplant.59.032607.092839 18444906

[B6] Limpens E Franken C Smit P Willemse J Bisseling T Geurts R LysM domain receptor kinases regulating rhizobial Nod factor-induced infection Science 2003 302 630 633 10.1126/science.1090074 12947035

[B7] Radutoiu S Madsen LH Madsen EB Felle HH Umehara Y Grønlund M Sato S Nakamura Y Tabata S Sandal N Stougaard J Plant recognition of symbiotic bacteria requires two LysM receptor-like kinases Nature 2003 425 585 592 10.1038/nature02039 14534578

[B8] Madsen EB Madsen LH Radutoiu S Olbryt M Rakwalska M Szczyglowski K Sato S Kaneko T Tabata S Sandal N Stougaard J A receptor kinase gene of the LysM type is involved in legume perception of rhizobial signals Nature 2003 425 637 640 10.1038/nature02045 14534591

[B9] Gough C Cullimore J Lipo-chitooligosaccharide signaling in endosymbiotic plant-microbe interactions Mol Plant Microbe Interact 2011 24 867 878 10.1094/MPMI-01-11-0019 21469937

[B10] Ané JM Kiss GB Riely BK Penmetsa RV Oldroyd GE Ayax C Lévy J Debellé F Baek JM Kalo P Rosenberg C Roe BA Long SR Dénarié J Cook DR Medicago truncatula DMI1 required for bacterial and fungal symbioses in legumes Science 2004 303 1364 1367 10.1126/science.1092986 14963334

[B11] Esseling JJ Lhuissier FG Emons AM A nonsymbiotic root hair tip growth phenotype in NORK-mutated legumes: implications for nodulation factor-induced signaling and formation of a multifaceted root hair pocket for bacteria Plant Cell 2004 16 933 944 10.1105/tpc.019653 15031407 PMC412867

[B12] Riely BK Lougnon G Ané JM Cook DR The symbiotic ion channel homolog DMI1 is localized in the nuclear membrane of Medicago truncatula roots Plant J 2007 49 208 216 10.1111/j.1365-313X.2006.02957.x 17173544

[B13] Charron D Pingret JL Chabaud M Journet EP Barker DG Pharmacological evidence that multiple phospholipid signaling pathways link Rhizobium nodulation factor perception in Medicago truncatula root hairs to intracellular responses, including Ca2+ spiking and specific ENOD gene expression Plant Physiol 2004 136 3582 3593 10.1104/pp.104.051110 15489277 PMC527157

[B14] Peleg-Grossman S Volpin H Levine A Root hair curling and Rhizobium infection in Medicago truncatula are mediated by phosphatidylinositide-regulated endocytosis and reactive oxygen species J Exp Bot 2007 58 1637 1649 10.1093/jxb/erm013 17420174

[B15] Foucher F Kondorosi E Cell cycle regulation in the course of nodule organogenesis in Medicago Plant Mol Biol 2000 43 773 786 10.1023/A:1006405029600 11089876

[B16] Roudier F Fedorova E Lebris M Lecomte P Györgyey J Vaubert D Horvath G Abad P Kondorosi A Kondorosi E The Medicago species A2-type cyclin is auxin regulated and involved in meristem formation but dispensable for endoreduplication-associated developmental programs Plant Physiol 2003 131 1091 1103 10.1104/pp.102.011122 12644661 PMC166874

[B17] Kondorosi E Redondo-Nieto M Kondorosi A Ubiquitin-mediated proteolysis. To be in the right place at the right moment during nodule development Plant Physiol 2005 137 1197 1204 10.1104/pp.105.060004 15824282 PMC1088313

[B18] Mathesius U Schlaman HR Spaink HP Sautter C Rolfe BG Djordjevic MA Auxin transport inhibition precedes root nodule formation in white clover roots and is regulated by flavonoids and derivatives of chitin oligosaccharides Plant J 1998 14 23 34 10.1046/j.1365-313X.1998.00090.x 15494052

[B19] Oldroyd GE Murray JD Poole PS Downie JA The rules of engagement in the legume-rhizobial symbiosis Annu Rev Genet 2011 45 119 144 10.1146/annurev-genet-110410-132549 21838550

[B20] Frugier F Kosuta S Murray JD Crespi M Szczyglowski K Cytokinin: secret agent of symbiosis Trends Plant Sci 2008 13 115 120 10.1016/j.tplants.2008.01.003 18296104

[B21] Boot KJM van Brussel AAN Tak T Spaink HP Kijne JW Lipochitin oligosaccharides from Rhizobium leguminosarum bv. viciae reduce auxin transport capacity in Vicia sativa subsp. nigra roots Mol Plant Microbe Interact 1999 12 839 844 10.1094/MPMI.1999.12.10.839

[B22] van Noorden GE Ross JJ Reid JB Rolfe BG Mathesius U Defective long-distance auxin transport regulation in the Medicago truncatula super numeric nodules mutant Plant Physiol 2006 140 1494 1506 10.1104/pp.105.075879 16489131 PMC1435797

[B23] Wasson AP Pellerone FI Mathesius U Silencing the flavonoid pathway in Medicago truncatula inhibits root nodule formation and prevents auxin transport regulation by rhizobia Plant Cell 2006 18 1617 1629 10.1105/tpc.105.038232 16751348 PMC1488924

[B24] Tirichine L Sandal N Madsen LH Radutoiu S Albrektsen AS Sato S Asamizu E Tabata S Stougaard J A gain-of-function mutation in a cytokinin receptor triggers spontaneous root nodule organogenesis Science 2007 315 104 107 10.1126/science.1132397 17110537

[B25] Murray JD Karas BJ Sato S Tabata S Amyot L Szczyglowski K A cytokinin perception mutant colonized by Rhizobium in the absence of nodule organogenesis Science 2007 315 101 104 10.1126/science.1132514 17110535

[B26] Gonzalez-Rizzo S Crespi M Frugier F The Medicago truncatula CRE1 cytokinin receptor regulates lateral root development and early symbiotic interaction with Sinorhizobium meliloti Plant Cell 2006 18 2680 2693 10.1105/tpc.106.043778 17028204 PMC1626621

[B27] Murray JD Invasion by invitation: rhizobial infection in legumes Mol Plant Microbe Interact 2011 24 631 639 10.1094/MPMI-08-10-0181 21542766

[B28] Marsh JF Rakocevic A Mitra RM Brocard L Sun J Eschstruth A Long SR Schultze M Ratet P Oldroyd GE Medicago truncatula NIN is essential for rhizobial-independent nodule organogenesis induced by autoactive calcium/calmodulin-dependent protein kinase Plant Physiol 2007 144 324 335 10.1104/pp.106.093021 17369436 PMC1913781

[B29] Haney CH Long SR Plant flotillins are required for infection by nitrogen-fixing bacteria Proc Natl Acad Sci USA 2010 107 478 483 10.1073/pnas.0910081107 20018678 PMC2806772

[B30] Lefebvre B Timmers T Mbengue M Moreau S Hervé C Tóth K Bittencourt-Silvestre J Klaus D Deslandes L Godiard L Murray JD Udvardi MK Raffaele S Mongrand S Cullimore J Gamas P Niebel A Ott T A remorin protein interacts with symbiotic receptors and regulates bacterial infection Proc Natl Acad Sci USA 2010 107 2343 2348 10.1073/pnas.0913320107 20133878 PMC2836688

[B31] Mergaert P Nikovics K Kelemen Z Maunoury N Vaubert D Kondorosi A Kondorosi E A novel family in Medicago truncatula consisting of more than 300 nodule-specific genes coding for small, secreted polypeptides with conserved cysteine motifs Plant Physiol 2003 132 161 173 10.1104/pp.102.018192 12746522 PMC166962

[B32] Gamas P Niebel Fde C Lescure N Mol Plant Microbe Interact 1996 9 233 242 10.1094/MPMI-9-0233 8634476

[B33] El Yahyaoui F Küster H Amor BB Hohnjec N Pühler A Becker A Gouzy J Vernié T Gough C Niebel A Godiard L Gamas P Expression profiling in Medicago truncatula identifies more than 750 genes differentially expressed during nodulation, including many potential regulators of the symbiotic program Plant Physiol 2004 136 3159 3176 10.1104/pp.104.043612 15466239 PMC523376

[B34] Pii Y Astegno A Peroni E Zaccardelli M Pandolfini T Crimi M The Medicago truncatula N5 gene encoding a root-specific lipid transfer protein is required for the symbiotic interaction with Sinorhizobium meliloti Mol Plant Microbe Interact 2009 22 1577 1587 10.1094/MPMI-22-12-1577 19888823

[B35] Pii Y Pandolfini T Crimi M Signaling LTPs: A new plant LTPs sub-family? Plant Signal Behav 2010 5 594 597 10.4161/psb.11499 PMC7080482 20404561

[B36] Higo K Ugawa Y Iwamoto M Korenaga T Plant cis-acting regulatory DNA elements (PLACE) database Nucleic Acids Res 1999 27 297 300 10.1093/nar/27.1.297 9847208 PMC148163

[B37] Boisson-Dernier A Andriankaja A Chabaud M Niebel A Journet EP Barker DG de Carvalho-Niebel F MtENOD11 gene activation during rhizobial infection and mycorrhizal arbuscule development requires a common AT-rich-containing regulatory sequence Mol Plant Microbe Interact 2005 18 1269 1276 10.1094/MPMI-18-1269 16478046

[B38] Vieweg MF Fruhling M Quandt HJ Heim U Baumlein H Puhler A Kuster H Andreas MP he promoter of the Vicia faba L. leghemoglobin gene VfLb29 is specifically activated in the infected cells of root nodules and in the arbuscule-containing cells of mycorrhizal roots from different legume and non legume plants Mol Plant Microbe Interact 2004 17 62 69 10.1094/MPMI.2004.17.1.62 14714869

[B39] Fehlberg V Vieweg MF Dohmann EM Hohnjec N Puhler A Perlick AM Kuster H The promoter of theleghaemoglobingeneVfLb29: functional analysis and identification of modules necessary for its activation in the infected cells of root nodules and in the arbuscule-containing cells of mycorrhizal roots. J Exp Bot 2005 56 799 806 10.1093/jxb/eri074 15668224

[B40] Hirsch S Kim J Muñoz A Heckmann AB Downie JA Oldroyd GE GRAS proteins form a DNA binding complex to induce gene expression during nodulation signaling in Medicago truncatula Plant Cell 2009 21 545 557 10.1105/tpc.108.064501 19252081 PMC2660633

[B41] Ross EJ Stone JM Elowsky CG Arredondo-Peter R Klucas RV Sarath G Activation of the Oryza sativa non-symbiotic haemoglobin-2 promoter by the cytokinin-regulated transcription factor, ARR1 J Exp Bot 2004 55 1721 1731 10.1093/jxb/erh211 15258171

[B42] Ballas N Wong LM Theologis A Identification of the auxin-responsive element, AuxRE, in the primary indoleacetic acid-inducible gene, PS-IAA4/5, of pea (Pisum sativum) J Mol Biol 1993 233 580 596 10.1006/jmbi.1993.1537 8411166

[B43] Doherty D Leigh JA Glazebrook J Walker GC Rhizobium meliloti mutants that overproduce the R. meliloti acidic calcofluor-binding exopolysaccharide J Bacteriol 1988 170 4249 4256 2842307 10.1128/jb.170.9.4249-4256.1988 PMC211434

[B44] Charon C Sousa C Crespi M Kondorosi A Alteration of enod40 expression modifies Medicago truncatula root nodule development induced by Sinorhizobium meliloti Plant Cell 1999 11 1953 1966 10521525 10.1105/tpc.11.10.1953 PMC144109

[B45] Imin N Goffard N Nizamidin M Rolfe BG Genome-wide transcriptional analysis of super-embryogenic Medicago truncatula explant cultures BMC Plant Biol 2008 8 110 10.1186/1471-2229-8-110 18950541 PMC2605756

[B46] He J Benedito VA Wang M Murray JD Zhao PX Tang Y Udvardi MK The Medicago truncatula gene expression atlas web server BMC Bioinformatics 2009 10 441 10.1186/1471-2105-10-441 20028527 PMC2804685

[B47] Cook D Dreyer D Bonnet D Howell M Nony E VandenBosch K Transient induction of a peroxidase gene in Medicago truncatula precedes infection by Rhizobium meliloti Plant Cell 1995 7 43 55 7696879 10.1105/tpc.7.1.43 PMC160763

[B48] Journet EP El-Gachtouli N Vernoud V de Billy F Pichon M Dedieu A Arnould C Morandi D Barker DG Gianinazzi-Pearson V Medicago truncatula ENOD11: a novel RPRP-encoding early nodulin gene expressed during mycorrhization in arbuscule-containing cells. Mol Plant Microbe Interact 2001 14 737 748 10.1094/MPMI.2001.14.6.737 11386369

[B49] Tadege M Wen J He J Tu H Kwak Y Eschstruth A Cayrel A Endre G Zhao PX Chabaud M Ratet P Mysore K Large scale insertional mutagenesis using Tnt1 retrotransposon in the model legume Medicago truncatula Plant J 2008 54 335 347 10.1111/j.1365-313X.2008.03418.x 18208518

[B50] Wais RJ Galera C Oldroyd GE Catoira R Penmetsa RV Cook D Gough C Denarié J Long SR Genetic analysis of calcium spiking responses in nodulation mutants of Medicago truncatula Proc Natl Acad Sci USA 2000 97 13407 13412 10.1073/pnas.230439797 11078514 PMC27237

[B51] den Hartog M Musgrave A Munnik T Nod factor-induced phosphatidic acid and diacylglycerol pyrophosphate formation: a role for phospholipase C and D in root hair deformation Plant J 2001 25 55 65 10.1046/j.1365-313x.2001.00931.x 11169182

[B52] Kelly MN Irving HR Nod factors activate both heterotrimeric and monomeric G-proteins in Vigna unguiculata (L.) Walp Planta 2003 216 674 685 12569410 10.1007/s00425-002-0900-8

[B53] Pingret JL Journet EP Barker DG Rhizobium Nod factor signalling: evidence for a G protein-mediated transduction mechanism Plant Cell 1998 10 659 671 9596628 10.1105/tpc.10.5.659 PMC144376

[B54] Ke D Fang Q Chen C Zhu H Chen T Chang X Yuan S Kang H Ma L Hong Z Zhang Z The small GTPase ROP6 interacts with NFR5 and is involved in nodule formation in Lotus japonicus Plant Physiol 2012 159 131 143 10.1104/pp.112.197269 22434040 PMC3375957

[B55] Irving HR Boukli NM Kelly MN Broughton WJ Ridge RW, Emons AM Nod-factors in symbiotic development of root hairs Root Hairs: Cell and Molecular Biology 2000 Tokyo: Springer-Verlag 241 265

[B56] Kelly MN Irving HR Nod factors stimulate plasma membrane delimited phospholipase C activity in vitro Physiol Plant 2001 113 461 468 10.1034/j.1399-3054.2001.1130404.x

[B57] Kelly-Skupek MN Irving HR Pharmacological evidence for activation of phospholipid and small GTP binding protein signalling cascades by Nod factors Plant Physiol Biochem 2006 44 132 142 10.1016/j.plaphy.2006.03.004 16647267

[B58] Dhonukshe P Laxalt AM Goedhart J Gadella TW Munnik T Phospholipase D activation correlates with microtubule reorganization in living plant cells Plant Cell 2003 15 2666 2679 10.1105/tpc.014977 14508002 PMC280570

[B59] Munnik T Arisz SA De Vrije T Musgrave A G Protein activation stimulates Phospholipase D signaling in plants Plant Cell 1995 7 2197 2210 12242371 10.1105/tpc.7.12.2197 PMC161073

[B60] Stracke S Kistner C Yoshida S Mulder L Sato S Kaneko T Tabata S Sandal N Stougaard J Szczyglowski K Parniske M A plant receptor-like kinase required for both bacterial and fungal symbiosis Nature 2002 417 959 962 10.1038/nature00841 12087405

[B61] Schauser L Roussis A Stiller J Stougaard J A plant regulator controlling development of symbiotic root nodules Nature 1999 402 191 195 10.1038/46058 10647012

[B62] Zanetti ME Blanco FA Beker MP Battaglia M Aguilar OM A C subunit of the plant nuclear factor NF-Y required for rhizobial infection and nodule development affects partner selection in the common bean–Rhizobium etli symbiosis Plant Cell 2010 22 4142 4157 10.1105/tpc.110.079137 21139064 PMC3027164

[B63] Soto MJ Domínguez-Ferreras A Pérez-Mendoza D Sanjuán J Olivares J Mutualism versus pathogenesis: the give-and-take in plant-bacteria interactions Cell Microbiol 2009 11 381 388 10.1111/j.1462-5822.2009.01282.x 19134114

[B64] Mortier V Holsters M Goormachtig S Never too many? How legumes control nodule numbers Plant Cell Environ 2012 35 245 258 10.1111/j.1365-3040.2011.02406.x 21819415

[B65] Brechenmacher L Kim MY Benitez M Li M Joshi T Calla B Lee MP Libault M Vodkin LO Xu D Lee SH Clough SJ Stacey G Transcription profiling of soybean nodulation by Bradyrhizobium japonicum Mol Plant Microbe Interact 2008 21 631 645 10.1094/MPMI-21-5-0631 18393623

[B66] Vasse J de Billy F Truchet G Abortion of infection during the Rhizobium meliloti-alfalfa symbiotic interactions is accompanied by a hypersensitive reaction Plant J 1993 4 555 566 10.1046/j.1365-313X.1993.04030555.x

[B67] Tellstrom V Usadel B Thimm O Stitt M Kuster H Niehaus K The lipopolysaccharide of Sinorhizobium meliloti suppresses defense-associated gene expression in cell cultures of the host plant Medicago truncatula Plant Physiol 2007 143 825 837 17220366 10.1104/pp.106.090985 PMC1803732

[B68] Popp C Ott T Regulation of signal transduction and bacterial infection during root nodule symbiosis Curr Opin Plant Biol 2011 14 458 467 10.1016/j.pbi.2011.03.016 21489860

[B69] Hirsch AM Bhuvaneswari TV Torrey JG Bisseling T Early nodulin genes are induced in alfalfa root outgrowths elicited by auxin transport inhibitors Proc Natl Acad Sci USA 1989 86 1244 1248 10.1073/pnas.86.4.1244 16594017 PMC286664

[B70] Ferraioli S Tate R Rogato A Chiurazzi M Patriarca EJ Development of ectopic roots from abortive nodule primordia Mol Plant Microbe Interact 2004 17 1043 1050 10.1094/MPMI.2004.17.10.1043 15497397

[B71] Yeats TH Rose JKC The biochemistry and biology of extracellular plant lipid-transfer proteins (LTPs) Protein Sci 2008 17 191 198 10.1110/ps.073300108 18096636 PMC2222726

[B72] Chae K Lord EM Pollen tube growth and guidance: roles of small, secreted proteins Ann Bot 2011 108 627 636 10.1093/aob/mcr015 21307038 PMC3170145

[B73] Maldonado AM Dixon PA Lamb CJ Cameron RK A putative lipid transfer protein involved in systemic resistance signalling in Arabidopsis Nature 2002 419 399 403 10.1038/nature00962 12353036

[B74] Chae K Kieslich CA Morikis D Kim SC Lord EM A gain-of-function mutation of Arabidopsis lipid transfer protein 5 disturbs pollen tube tip growth and fertilization Plant Cell 2009 21 3902 3914 10.1105/tpc.109.070854 20044438 PMC2814499

[B75] Meade HM Long SR Ruvkun GB Brown SE Ausubel FM Physical and genetic characterization of symbiotic and auxotrophic mutants of Rhizobium meliloti induced by transposon Tn5 mutagenesis J Bacteriol 1982 149 114 122 6274841 10.1128/jb.149.1.114-122.1982 PMC216598

[B76] Quandt HJ Pühler A Broer I Transgenic root nodules ofVicia hirsuta: A fast and efficient system for the study of gene expression in indeterminate-type nodules. Mol Plant Microbe Interact 1993 6 699 706 10.1094/MPMI-6-699

[B77] Pii Y Crimi M Cremonese G Spena A Pandolfini T Auxin and nitric oxide control indeterminate nodule formation BMC Plant Biol 2007 7 21 10.1186/1471-2229-7-21 17488509 PMC1878477

[B78] Engstrom EM Ehrhardt DW Mitra RM Long SR Pharmacological analysis of Nod factor-induced calcium spiking in Medicago truncatula. Evidence for the requirement of type IIA calcium pumps and phosphoinositide signaling Plant Physiol 2002 128 1390 1400 10.1104/pp.010691 11950987 PMC154266

[B79] Molesini B Pandolfini T Pii Y Korte A Spena A Arabidopsis thaliana AUCSIA-1 regulates auxin biology and physically interacts with a kinesin-related protein PLoS One 2012 7 e41327 10.1371/journal.pone.0041327 22911780 PMC3401106

[B80] Bevan M Binary Agrobacterium vectors for plant transformation Nucleic Acids Res 1984 12 8711 8721 10.1093/nar/12.22.8711 6095209 PMC320409

[B81] Oldroyd GE Long SR Identification and characterization of nodulation-signaling pathway 2, a gene of Medicago truncatula involved in Nod factor signaling Plant Physiol 2003 131 1027 1032 10.1104/pp.102.010710 12644655 PMC166868

[B82] Veereshlingam H Haynes JG Penmetsa RV Cook DR Sherrier DJ Dickstein R nip, a symbiotic Medicago truncatula mutant that forms root nodules with aberrant infection threads and plant defense-like response Plant Physiol 2004 136 3692 3702 10.1104/pp.104.049064 15516506 PMC527167

[B83] Boisson-Dernier A Chabaud M Garcia F Becard G Rosenberg C Barker DG Agrobacterium rhizogenes-transformed roots of Medicago truncatula for the study of nitrogen-fixing and endomycorrhizal symbiotic associations Mol Plant Microbe Interact 2001 14 695 700 10.1094/MPMI.2001.14.6.695 11386364

[B84] DuBois M Gilles KA Hamilton JK Rebers PA Smith F Colorimetric method for determination of sugars and related substances Anal Chem 1956 28 350 356 10.1021/ac60111a017

[B85] Molesini B Pandolfini T Rotino GL Dani V Spena A Aucsia gene silencing causes parthenocarpic fruit development in tomato Plant Physiol 2009 149 534 548 10.1104/pp.108.131367 18987210 PMC2613741

