# Supplementary figures and images for: The non-specific lipid transfer protein N5 of Medicago truncatula is implicated in epidermal stages of rhizobium-host interaction
Source: BMC Plant Biol. 2012 Dec 7;12:233. doi: 10.1186/1471-2229-12-233 (PMC3564872; doi:10.1186/1471-2229-12-233)

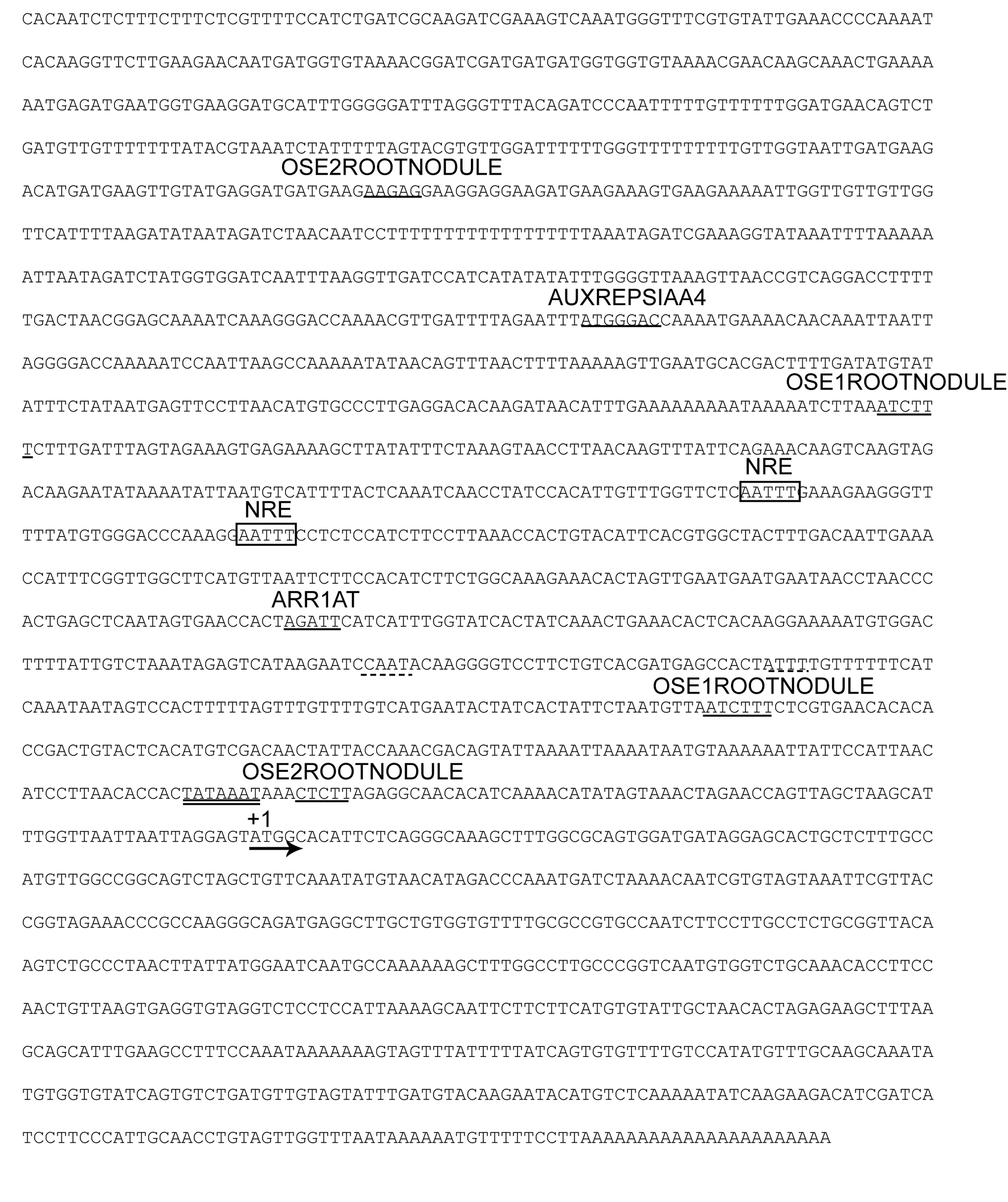

Supplement: Additional file 1 — Nucleotide sequence of the putative MtN5 promoter and MtN5 open reading frame. The beginning of the ORF is identified by +1. The region analysed as the putative promoter encompasses 1.54 kb upstream the initiation codon. TATA box and CAAT box are double underlined and underlined with dashed line, respectively. The other motifs highlighted are listed as follow: OSE1ROOTNODULE (AAAGAT occurring in antisense orientation), OSE2ROOTNODULE (AAGAG occurring in both sense and antisense orientations), NRE (AATTT) , ARR1AT (AGATT) and AUXREPSIAA4 (GTCCCAT occurring in antisense orientation). [file 1471-2229-12-233-S1.tiff]

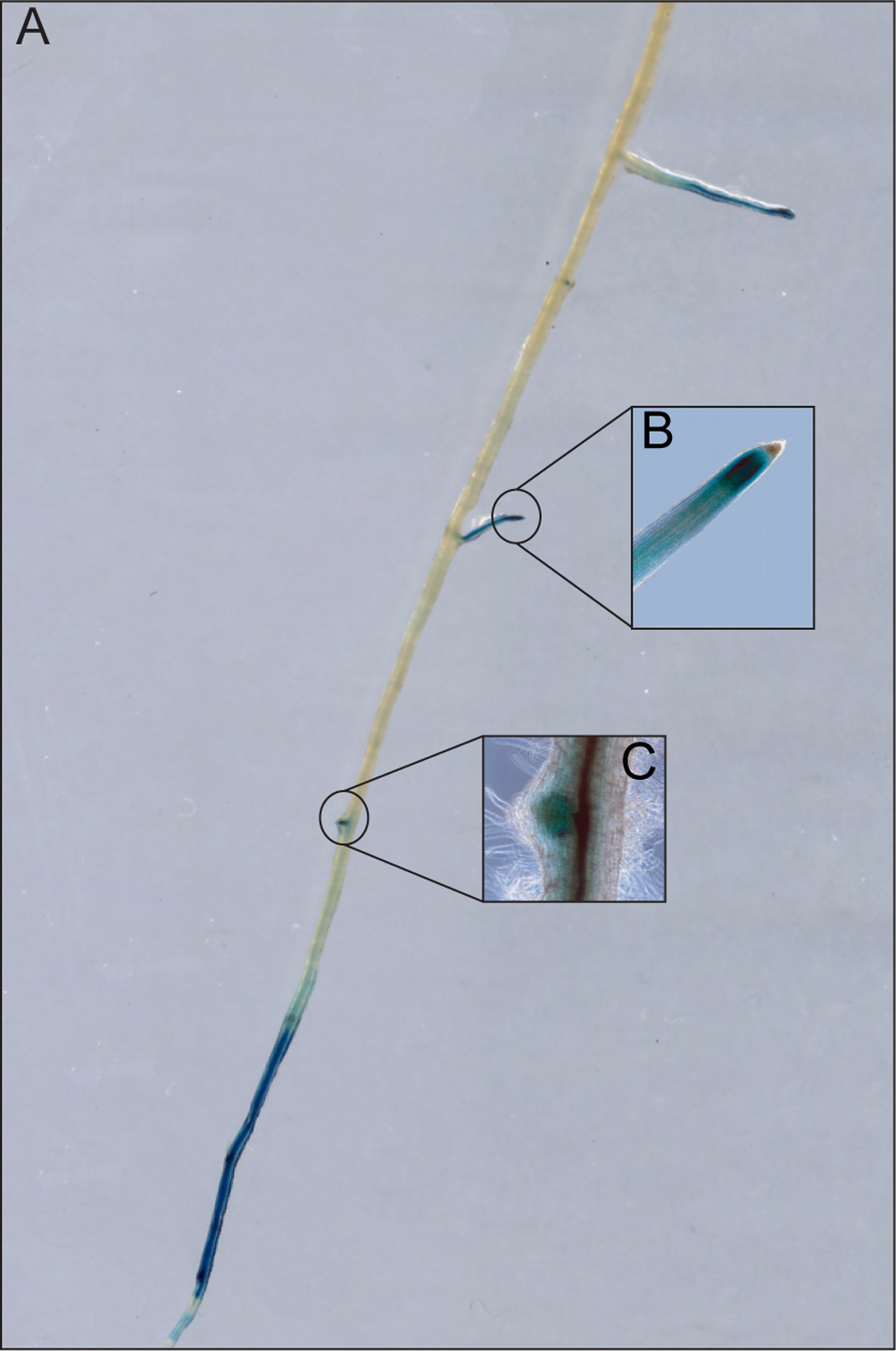

Supplement: Additional file 2 — MtN5 promoter activity in non-inoculated M. truncatula root tissue. A. Representative GUS staining pattern in M. truncatula transgenic adventitious roots harbouring the MtN5::GUS construct. Insets show the representative GUS staining pattern at the lateral root apex (B) and at the lateral root emergence (C), respectively. [file 1471-2229-12-233-S2.tiff]

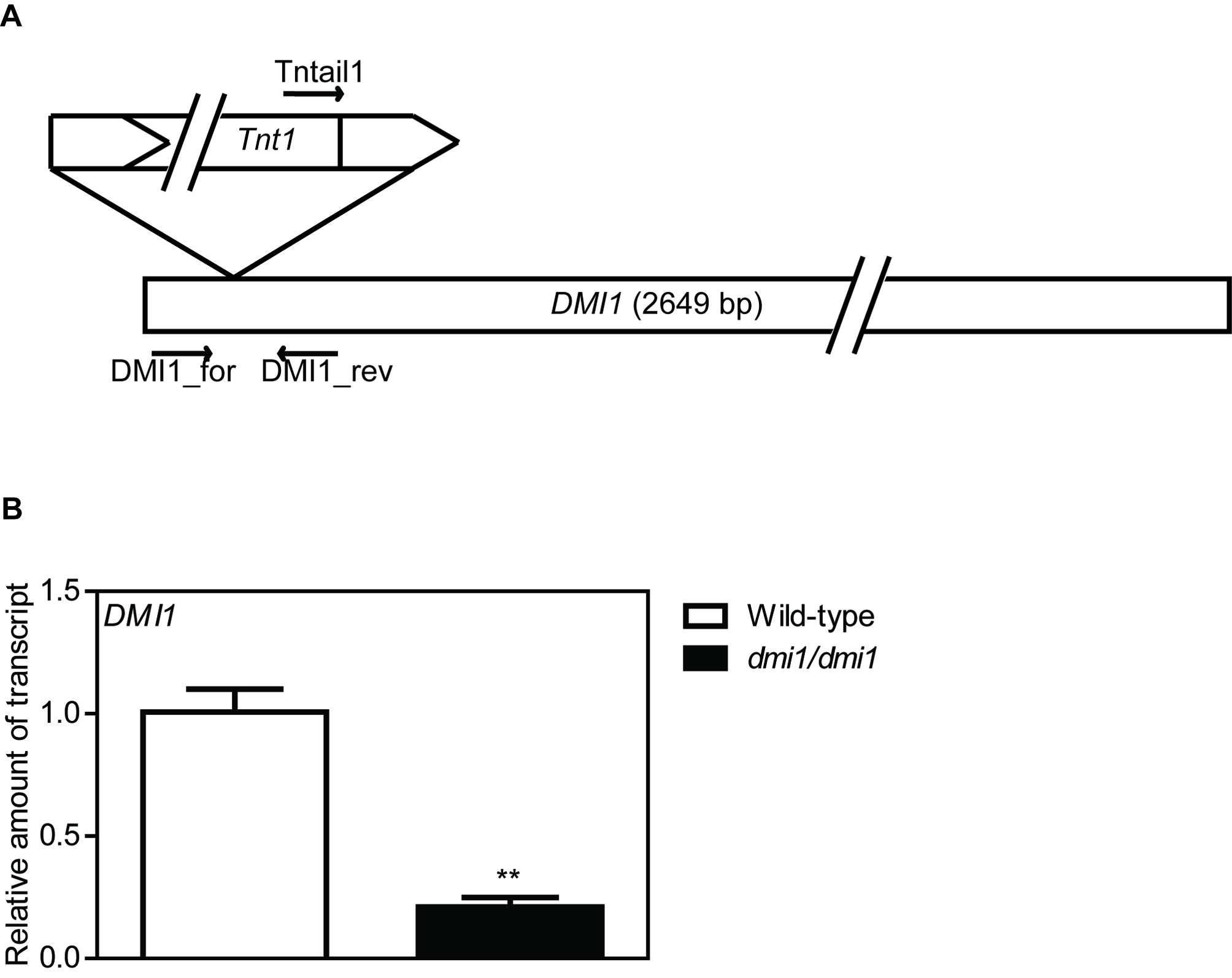

Supplement: Additional file 3 — Description of NF4257 insertional mutant and determination of DMI1 expression in the mutated background. A. Schematic drawing of the Tnt1 insertion and representation of the oligonucleotides used for the genetic characterization of the insertional line. B. qRT-PCR analysis of DMI1 expression in M. truncatula R108 wild-type and NF4257 roots inoculated with S. meliloti. The expression data were normalized to an internal actin control. The relative expression ratio was calculated using inoculated wild type roots as calibrator sample. The values reported are means ± SE (n=3). Student’s t test was applied. **, P < 0.01. [file 1471-2229-12-233-S3.tiff]

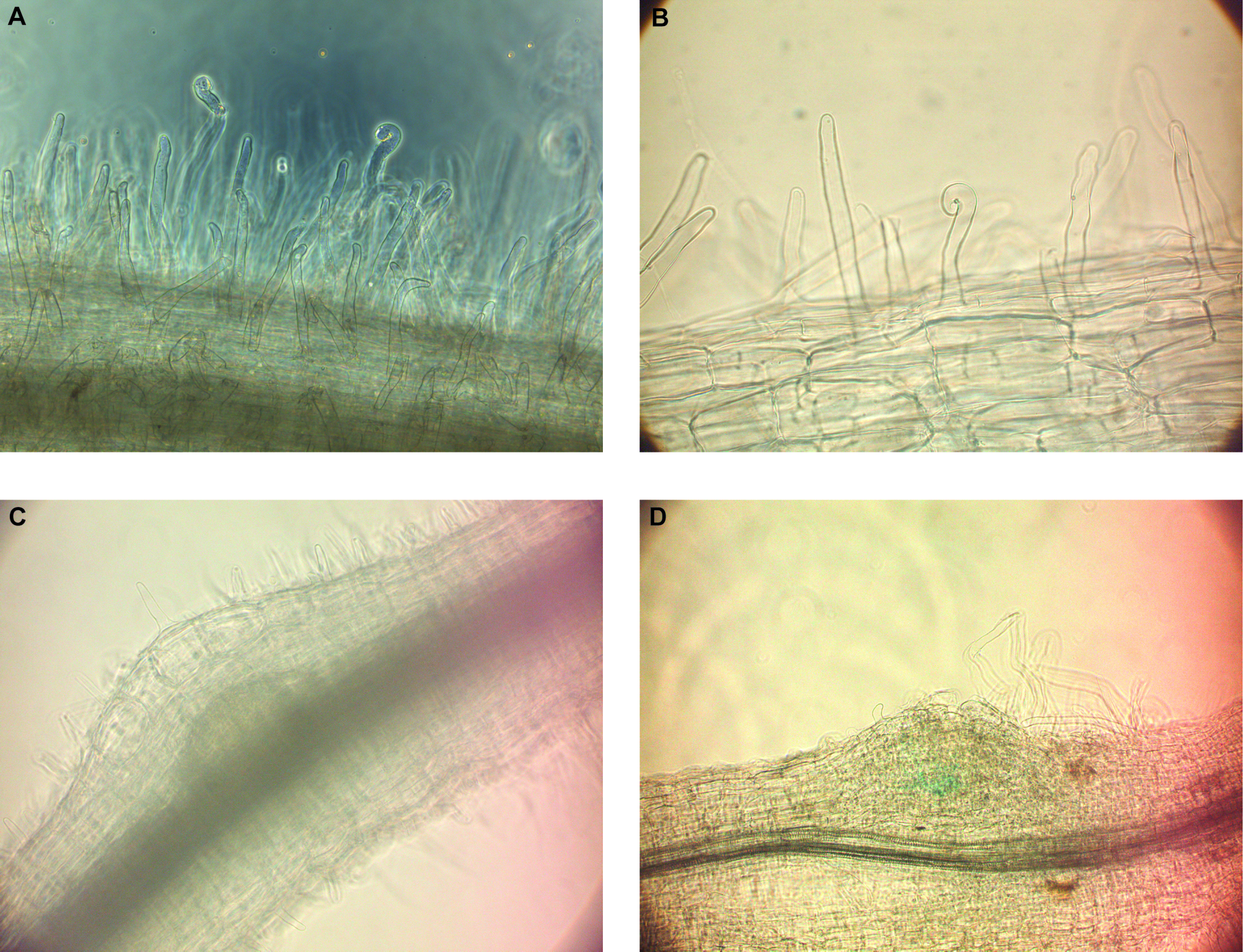

Supplement: Additional file 4 — Root hair deformations and nodule primordia in MtN5-silenced and control roots. A-B. Representative micrographs of root hair curling in M. truncatula roots inoculated with S. meliloti. A. Transgenic adventitious root bearing the MtN5hp gene construct. B. Transgenic adventitious root bearing an empty T-DNA (Control sample). C-D. Representative micrographs of root nodule primordia in M. truncatula roots inoculated with S. meliloti carrying the pXLGD4 plasmid. The presence of rhizobia within the primordia was highlighted through the staining for the β-galactosidase activity. C. Nodule primordium generated on transgenic adventitious root bearing the MtN5hp gene construct. D. Nodule primordium generated on transgenic adventitious root bearing an empty T-DNA (Control sample). [file 1471-2229-12-233-S4.tiff]
