# Supplementary material for: The non-specific lipid transfer protein N5 of Medicago truncatula is implicated in epidermal stages of rhizobium-host interaction
Source: BMC Plant Biol. 2012 Dec 7;12:233. doi: 10.1186/1471-2229-12-233 (PMC3564872; doi:10.1186/1471-2229-12-233)
Supplement: Additional file 5 — Primers used for RT-PCR. List of the oligonucleotides used as primers in the qRT-PCR experiments. [file 1471-2229-12-233-S5.doc]

| **Gene** | **Forward (5’-3’)** | **Reverse (5’-3’)** |
| --- | --- | --- |
| ***MtN5*** | CTGCGGTTACAAGTCTGCCCTAAC | GCGGATCCTTAACAGTTGGAAGGTGTTTG |
| ***ACTIN*** | AGATGCTGAGGATATTCAAC | GTATGACGAGGTCGGCCAAC |
| ***ENOD11*** | ATGGCTTCCTTTTTCTTGTACTCGC | TGTGTAAATTGGATTTGGAGGCAT |
| ***MtNIN*** | ATGGAATATGGTGGTGGGTTAGTG | TTCAATCCAGCAACCTTCTCCCAA |
| ***FLOT4*** | TGCGTCTGCTAATGCTTTCTGTG | CCGAAGTTGAGGCTGCCAAAG |
| ***DMI1*** | ATGGGGACATCAGTAATATGTAGA | CTGATCTGCATTTTCGTCCGCAGC |

List of the oligonucleotides used as primers in the qRT-PCR experiments.
